# Preface to the Special Edition on Femtochemistry and “The Hamburg Conference on Femtochemistry 2015 (FEMTO12)”

**DOI:** 10.1063/1.4961613

**Published:** 2016-08-24

**Authors:** Terry Mullins, Jochen Küpper

**Affiliations:** 1 Center for Free-Electron Laser Science, DESY, Notkestrasse 85, 22607 Hamburg, Germany; 2 Department of Physics, University of Hamburg, Luruper Chaussee 149, 22761 Hamburg, Germany; 3 The Hamburg Center for Ultrafast Imaging, University of Hamburg, Luruper Chaussee 149, 22761 Hamburg, Germany

This special issue is dedicated to the memory of Dr. Ahmed Zewail, the founder of the field and the public-keynote speaker of the *Leuchtturm* (Tower of Light) Lecture (https://www.femto12.org/leuchtturm) at FEMTO12.

Femtochemistry addresses the very nature of the chemical bond. It aims at unraveling the ultrafast dynamics of bond forming and breaking. Molecules and atoms are the building blocks of nature, and their chemical dynamics define the evolution of our world. Chemical bonds are made by valence electrons and have energies of a few electron volts (eV). Chemical reactions are mostly initiated by heat. The huge driving forces of chemical reactions are the strong intramolecular Coulomb forces, mediated by valence electron and nuclear dynamics. Sometimes, light is used to trigger chemical reactions, often to mimick the sudden availability of thermal energy. Because light can be provided in ultrashort pulses with very high temporal precision, such pulses provide a well defined starting point of the chemical reaction and allow to clock the ongoing subsequent chemical dynamics.^1^ The resulting high temporal resolution makes ultrashort laser pulses a superb tool to investigate the ultrafast steps of chemical processes, i.e., femtochemistry.

Through this approach to ultrafast chemical processes, femtochemistry crosses the traditional boundaries of physics, chemistry, and biology. Within its scope, a variety of questions arise: How do the building blocks of chemistry, such as atoms, molecules, and light, interact with one another at the most fundamental level and on the shortest time scales to drive both simple and complex systems from initial to final states? What is the role of the transition state^2^ and how can we directly observe it? How can we understand increasingly complex systems? How do the most rapid dynamics of the smallest sub-systems and particles initiate large scale changes in macroscopic systems on long timescales? How much correlation is contained in that extension? Can biological systems be understood in terms of their quantum properties? In how far can all these processes be controlled and driven to tailor desired processes and materials?

New kids on the block for the investigation of these properties are the recently introduced short-wavelength free-electron lasers and ultrashort-pulse electron sources, which aim at recording so-called molecular movies with femtosecond temporal and atomic-resolution (100 pm) spatial resolution. Their application in femtochemistry and femtobiology was highlighted elsewhere.^3–5^

This special issue was initiated together with the Hamburg Conference on Femtochemistry (FEMTO12), which was the twelfth in a series of conferences that was started in Berlin in 1993. FEMTO12 was held in Hamburg-Bahrenfeld on 12–17 July 2015 and provided a platform for rich discussions on the ongoing experimental and theoretical work to disentangle and understand ultrafast molecular processes related to valence electron dynamics. This includes studies of these effects in molecules and clusters, liquids and solutions, biological systems, interfaces and surfaces, nanosystems, and polymers and condensed matter. The eleven papers in this issue highlight some of the wonderful research presented and the constructive discussions had at the conference.

DNA is arguably one of the most important biological molecules. In nature, it is exclusively found in a hydrated environment and its interactions with water are, therefore, of great importance. The dynamics of hydrated DNA oligomers are investigated by Guchhait *et al*.^6^ Energy transport is an important process, which naturally lends itself to laser control techniques. Brüning *et al*.^7^ investigate the controlled localization of energy in photo-excited aggregates. Thallmair *et al*.^8^ discuss the role of conical intersections, which become more prominent as complexity increases, in the ultrafast branching in the photoinduced bond cleavage process of diphenylmethyl derivatives. Catalytic reactions are extremely important for many practical applications. Kunnis *et al*.^9^ investigate the spin cross-over and ligation of the Fe(CO)_5_ complex in solution upon photoexcitation.

Bond breaking and bond rearrangement, the resulting transition states, as well as charge transfer processes belong to some of the most fundamental concepts in chemistry. Li *et al*.^10^ investigate the effect of the carrier envelope phase on dissociative photoionization of toluene. Stensitzki *et al*.^11^ characterize the photoreaction of hexacoordinated Al(tpfc-Br_8_)(py)_2_. Stensitzki *et al*.^12^ investigate photoisomerization products and timescales in channelrhodopsin-1. Boll *et al*.^13^ investigate ultrafast electron transfer in dissociating molecules. Kim *et al*.^14^ investigate the transition state structure of the ${\left[\text{Au}{\left(\text{CN}\right)}_{2}^{-}\right]}_{3}$ trimer upon photoexcitation. Imanbaew *et al*.^15^ investigate the cation and monoanion of fluorescein in the gas phase and observe signatures of differing excited state structures. Spatial structure can enormously affect dynamics and interactions. Li and Vendrell^16^ investigate protonated water clusters after extreme UV photoionization and find site-dependent correlated proton-electron hole dynamics.
